# Health-related quality of life in Chinese individuals with type 2 diabetes mellitus: a multicenter cross-sectional study

**DOI:** 10.1186/s12955-023-02183-1

**Published:** 2023-08-26

**Authors:** Zihuan Zeng, Xingli Wang, Yanhan Chen, Hengyu Zhou, Wenfen Zhu, Xiu Xiong, Jiao Tang, Qinghua Zhao

**Affiliations:** 1 Institute of Burn Research, The First Affiliated Hospital of Army Medical University, Chongqing, 400038 China; 2 School of Nursing, Chongqing Medical University, Chongqing, 400016 China; 3 Department of Dermatology, University-Town Hospital of Chongqing Medical University, Chongqing, China; 4 Department of Nursing, The First Affiliated Hospital of Chongqing Medical University, Chongqing, 400016 China

**Keywords:** Type 2 diabetes mellitus, Health-related quality of life, Fear of hypoglycemia, Fear of progression, Self-efficacy

## Abstract

**Background:**

Type 2 diabetes mellitus (T2DM) is a chronic and life-threatening disease. Health-related quality of life (HRQoL) is vital for individuals with T2DM. However, little is known about the impact of psychological stability factors on HRQoL among individuals with T2DM in mainland China.

**Methods:**

This multicenter cross-sectional study was conducted in five tertiary grade-A hospitals in Chongqing, China, from January to December 2019. A total of 385 individuals with T2DM were included by the convenient sample method. Fear of Progression (FOP) Questionnaire-short Form, Hypoglycemia Fear Survey II, diabetes-management self-efficacy scale, and EuroQol-5 Dimensions were used for data collection.

**Results:**

The mean age of the 385 individuals was 57.65 (SD = 15.15) years, three-quarters of whom had a high school or above education level. The participants in our study had moderate HRQoL and were more likely to have poor scores in the pain/discomfort dimension. The FOP level was moderate on average, and 23.1% of individuals suffered from psychological dysfunction. The participants had higher levels of fear of hypoglycemia (FOH) and self-efficacy (SE). Multiple steppage-regression analysis predicted that higher levels of FOP and FOH, reduced SE, older age, longer duration since diagnosis, lower educational attainment, higher levels of HbA1c, and living with comorbid conditions were related to lower HRQoL.

**Conclusion:**

This study showed that the HRQoL among Chinese T2DM patients may be impaired by increased FOP and FOH, decreased SE, and poor glycemic control. In addition, as the patient’s age and duration since diagnosis increase, their HRQoL further declines. We recommend improving HRQoL by encouraging individuals to attain more health education and resilience skills to enhance SE and reduce negative emotions among individuals with T2DM.

## Background

Type 2 diabetes mellitus (T2DM) is a metabolic disease characterized by chronic hyperglycemia caused by insulin resistance or insufficient insulin secretion due to various virulence factors. The International Diabetes Federation (IDF) Diabetes Atlas showed that the total number of diabetic people was 537.0 million globally in 2021, which was predicted to reach 783 million in 2045 [1]. In China, the prevalence of diabetes was 11.2%, and it is calculated that the number of individuals with diabetes would increase from 140.9 million in 2021 to 174.4 million in 2045 [1, 2]. Therefore, as the number of individuals with T2DM is so large worldwide, especially in China, further attention should be paid to this disease.

Quality of life (QoL) refers to an individual’s perception of his or her living conditions according to the existing value and cultural system, which is related to his or her expectations and living standards [3]. In addition, the genuine aim is to enhance individuals’ participation in medical decision-making by using measurement instruments based on patient perception to introduce the perspectives of individuals into clinical research [4]. Health-related quality of life (HRQoL), a concept of QoL relating solely to health aspects, is one of the most widely used methods to self-measure chronic disease management, alleviate disease impact on health, and monitor psychological, physiological, and social aspects of personal health [5]. Studies conducted by Grandy and Strain [6, 7] reported that the burden of T2DM (e.g., kidney disease, neuropathy, blindness microcirculation, and cardiovascular) could have a detrimental impact on the HRQoL of individuals in the long term. Therefore, researchers must pay more attention to the HRQoL of individuals with T2DM.

Previous studies have found that several sociodemographic characteristics (e.g., age, employment condition, educational attainment, and household income) of diabetic individuals might predict HRQoL [8, 9]. However, regional, economic, and cultural differences often lead to variation in the associated factors of HRQoL among T2DM individuals. Moreover, associated factors influencing the psychological stability of T2DM individuals, such as fear of progression (FOP), fear of hypoglycemia (FOH), and self-efficacy (SE), might have a significant impact on the HRQoL of individuals.

FOP, which has been defined as the fear of the progression of diseases with biopsychosocial consequences or recurrence [10], is one of the most common and influential mental burdens of individuals with chronic diseases. Extreme fear makes individuals pessimistic, leading to decreased treatment compliance and increased clinical risk of medical treatment and nursing, and may range from functional (e.g., thereby retaining treatment compliance) to dysfunctional levels [11]. There is convincing empirical evidence that FOP is prevalent in a variety of individuals suffering from chronic diseases [12]. For example, FOP has been shown to be associated with lowered HRQoL both in child and adult cancer patients. However, FOP is rarely used as a variable to study HRQoL in individuals with T2DM.

Hypoglycemia is a significant obstacle in controlling the plasma glucose levels of individuals with T2DM. One possible cause of hypoglycemia is drugs that cause unregulated exogenous (insulin) or endogenous hyperinsulinemia [13]. Despite hypoglycemic mortality rates being not clear, insulin and insulin secretagogue-induced hypoglycemia can be fatal in T2DM. Up to 10% of individuals with severe sulfonylureas hypoglycemia die. Hypoglycemia has also been found to cause severe cardiac arrhythmias [14]. Therefore, the discomfort brought by hypoglycemia and its potential threat to life can induce FOH in the patient [12]. Evidence shows that FOH harms clinical outcomes (e.g., microvascular complications) by making T2DM individuals engage in overcompensating behaviors (e.g., monitoring blood glucose frequently and reducing the use of insulin) [15–17]. At present, studies have mainly focused on the status and associated factors of FOH [18, 19]. However, a Spanish study on T2DM individuals with hypoglycemia showed that increased FOH negatively affected the HRQoL of T2DM individuals [20].

SE is described as one’s conception of their capability to execute and organize the progress of action required to deal with prospective status and stressors. SE conception influences the choices individuals make, their aspirations, how long they can persevere in a challenge, the amount of effort they put into realizing certain goals, the amount of stress, and their susceptibility to depression [21]. Better self-care activity and well-controlled glucose have demonstrated positive implications for SE and further improvements to the HRQoL of T2DM individuals [22].

In short, HRQoL is vital for T2DM individuals. However, little is known about the impact of FOP, FOH, and SE on HRQoL among individuals with T2DM in mainland China. Therefore, this study aimed to investigate HRQoL and identify its associated factors (e.g., FOP, FOP, and SE) among Chinese individuals with T2DM. The results of this study may enhance our understanding of the relationship between FOP, FOH, SE, and HRQoL, and help researchers find novel approaches to improve HRQoL among individuals with T2DM.

## Methods

### Study design and setting

This multicenter cross-sectional study was conducted in the departments of endocrinology of five tertiary grade-A hospitals in Chongqing, Southwest China. Individuals with T2DM were invited to take part in this study if they met the following inclusion criteria: (1) met WHO diagnostic criteria for T2DM; (2) were aged ≥ 18 years; (3) had primary school or above educational level; and (4) provided informed consent. We excluded medical personnel who had T2DM with pregnancy, malignant tumors, history or family history of mental illness, or other serious medical conditions that make it difficult to talk and fill out questionnaires. The sample size was estimated according to the equation $\mathrm{n}=\left[\mathrm{m}\left(10-15\right)\right]\left(1+20\mathrm{\%}\right)$, where m is the number of independent variables to be included in the planned linear regression [19, 23]. As there were 13 independent variables in this study, the calculated sample size had to be at least 156.

### Data collection

The data was collected by five investigators using multicenter convenience sampling between January 1 and December 31, 2019. Firstly, the investigators, who were majoring in endocrinology, had been given uniform guidance and training (e.g., questionnaire distribution and collection skills, and communication skills) before the survey. Then, individuals meeting the inclusion criteria were invited to participate in this study and signed a consent form. Subsequently, the investigators used unified guidance statements to guide the individuals to fill out the questionnaire by themselves. Questionnaires were administered and collected on the spot, and every questionnaire was checked item-by-item in the collecting process to ensure the authenticity and integrity of the data. Finally, 426 T2DM individuals were enrolled in this survey, and 385 individuals with complete data were eligible for data analysis. Therefore, the attrition rate $\left[\left(426-385\right)/426\right]$ of the T2DM individuals was 9.6%.

### Measures

#### EuroQol Five Dimensions (EQ-5D)

EQ-5D, developed by EuroQol Group, was used to measure HRQoL [24], which included a health status description system and a visual analogue scale (VAS). The health status description system consisted of five subscales: pain/discomfort, anxiety/depression, mobility, usual activities, and self-care. The health status description system could not be used to directly calculate the health effect value. Therefore, Chinese studies have adopted the time trade-off method and established the three-level EuroQol Five Dimensions (EQ-5D-3L) utility value integral system based on the Chinese population to obtain the health effect value for a total of 243 unique health status of the population [25], which contained three levels (i.e., 1 = no problems, 2 = some/moderate problems, and 3 = extreme problems) for each dimension. EQ-5D-3L provides utility values, of which 0.0 corresponds to death and 1.0 corresponds to full health. The EQ-VAS is a 20-cm vertical visual scale used to evaluate the overall health status of individuals. The top score of 100 represents the best health status in individuals’ minds, and the bottom score of 0 represents the worst health status. EQ-5D has good reported reliability and validity, with a Cronbach’s α coefficient of 0.846, and correlation coefficients (r) between the five dimensions in the health description system and VAS of − 0.475, − 0.415, − 0.517, − 0.494, and − 0.444, respectively [26].

#### Fear of Progression Questionnaire-Short Form (FOP-Q-SF)

FOP-Q-SF, which was adapted by Mehnert et al. [27] based on the Fear of Progression Questionnaire (FOP-Q) [28], was used to measure FOP. FOP-Q-SF is a single-dimensional scale with 12 items. All items were rated using a 5-point Likert scale ranging from never (one point) to very often (five points), with higher scores indicating a higher level of FOP. Individuals are considered to suffer from psychological dysfunction if their FoP-Q-SF score is ≥ 34 points. Cronbach’s α coefficient of the scale was 0.82, and the scale had a high correlation with FOP-Q (*r* = 0.92), showing good reliability and validity [27].

#### Hypoglycemia Fear Survey II (HFSII)

HFSII, the revised version of HFS, was used to measure FOH based on the experience of diabetic individuals in the last 6 months [15, 28, 29]. FOH was measured using the 33-item instrument and divided into a worry subscale (HFSII-WS) and a behavior subscale (HFSII-BS). Every item was rated using a 5-point Likert scale ranging from 0 to 4 points. Item scores were aggregated to form a total FOH score ranging from 0 to 132, with a higher score indicating a higher level of FOH [30]. HFSII showed good internal consistency with a Cronbach’s α coefficient of 0.904 and adequate content validity with a content validity index of 0.78–1.00 [31, 32].

#### Diabetes-Management SE Scale (DMSES)

DMSES was developed in 1999 by Bijl et al. [33] to assess the SE of diabetic individuals. Then, it was cross-culturally adapted into a Chinese version by Shu et al. [34]. The Chinese version of DMSES has 20 items divided into four subscales: medication, blood glucose monitoring, diet, and exercise. Every item was rated using an 11-point Likert scale ranging from 0 to 10 points. The total score of the Chinese version of DMSES ranges from 0 to 200 points, with higher scores indicating a higher level of diabetes-management SE. Its score index is equal to the actual score of the scale divided by the highest possible score of the scale multiplied by 100%, which is divided into three levels: high (≥ 80%), medium (40%–80%), and low (≤ 40%). DMSES showed adequate reliability and validity with a Cronbach’s α coefficient of 0.93, and a content validity index of 0.86 [34].

Sociodemographic and clinical characteristics such as age, gender, marital status, and educational attainment were collected using a sociodemographic and clinical data sheet. Diabetes-related complications consisted of diabetic cardiovascular events, diabetic brain lesions, diabetic nephropathy, diabetic peripheral neuropathy, diabetic foot, and diabetic retinopathy. Comorbid conditions comprised hypertension and hyperlipidemia.

#### Data analyses

This study used SPSS version 25.0 (IBM Corporation) for data analysis. Descriptive statistics were used to express the participants’ characteristics and study variables. Continuous variables conforming to the normal distribution were expressed as a mean (standard deviation [SD]); otherwise, median (inter-quartile range [IQR]) was used. Categorical variables were expressed using frequencies and percentages. For univariate analysis, Student’s *t*-test, one-way analysis of variance, or correlation tests were used to contrast the differences or correlations in the HRQoL of participants with different demographics, FOP, FOH, and SE. Subsequently, variables with *p* < 0.05 in the univariate analysis were entered into a multiple linear regression analysis with the stepwise method to analyze the associated factors of HRQoL. Notably, the following assumptions were tested before performing the multiple linear regression analysis: (1) scatter plots were drawn to test the linearity of the model; (2) a histogram was made to test the normality of the distribution of standardized residuals; (3) a Durbin-Watson value of approximately 2 to verify no autocorrelation in the residuals; (4) variance inflation factor < 10 to verify no multicollinearity between independent variables. If all assumptions were satisfied, multiple linear regression analysis was used to find the best-fit model for the associated factors of HRQoL; otherwise, the coefficient of determination (R^2^) was used to test the fitting degree of a multiple linear regression model, where *p* < 0.05 indicated statistical significance.

#### Ethical considerations

This study conformed to the provisions of the Declaration of Helsinki in 1995 (as revised in Brazil in 2013). Ethical approval was obtained from Chongqing Medical University Ethical Committee. Informed consent was obtained from all individuals who participated in the study.

## Results

### Sociodemographic and clinical characteristics of individuals with T2DM

Table 1 illustrates the sociodemographic and clinical characteristics of the study participants. Among the 385 participants, the mean age was 57.65 (SD = 15.15) years, and the female-to-male ratio was approximately 1:1. Most (86.8%) of the participants were married. Nearly three-quarters of the participants had a high school or above education level. More than 75.0% of the participants were hospitalized for the second time or more because of T2DM, but only 62.1% had received health education. More than half of the participants suffered from diabetes-related complications and comorbid conditions. The mean diabetic duration since diagnosis and hemoglobin A1c (HbA1c) were 9.93 (SD = 7.54) years and 10.31% (SD = 2.02%), respectively.

**Table 1:** Sociodemographic and clinical characteristics of individuals with T2DM (*n* = 385)

Characteristic	n	%	EQ-5D-3L			EQ-VAS		
			Mean (SD)	t/F/r	*P*	Mean (SD)	t/F/r	*P*
Age (years) Mean (standard deviation [SD]) = 57.65 (15.15)				** − 0.262**	**0.000**		** − 0.243**	**0.000**
Gender				− 0.211	0.833		1.170	0.243
Male	189	49.1	0.84 (0.21)			82.49 (11.57)		
Female	196	50.9	0.85 (0.20)			80.76 (11.35)		
Marital status				0.615	0.541		1.537	0.126
Married	334	86.8	0.85 (0.20)			82.01 (10.81)		
Unmarried	51	13.2	0.83 (0.23)			78.98 (14.20)		
Educational level				**9.181**	**0.000**		**9.387**	**0.000**
Primary school	105	27.3	0.80 (0.24)			75.47 (11.65)		
High school	184	47.8	0.84 (0.20)			82.20 (11.97)		
College or above	96	24.9	0.92 (0.14)			84.68 (10.56)		
Number of hospitalizations because of T2DM				**2.263**	**0.024**		**4.365**	**0.000**
First time	88	22.9	0.89 (0.18)			86.29 (10.72)		
Second time or more	297	77.1	0.83 (0.21)			79.53 (11.19)		
Receiving health education on diabetes				0.539	0.590		** − 3.963**	**0.000**
Yes	239	62.1	0.85 (0.21)			84.01 (11.08)		
No	146	37.9	0.84 (0.20)			78.34 (11.08)		
Diabetes-related complications				** − 4.554**	**0.000**		** − 2.895**	**0.004**
Yes	223	57.9	0.81 (0.22)			79.51 (11.95)		
No	162	42.1	0.90 (0.16)			83.72 (10.45)		
Comorbid conditions				** − 2.076**	**0.039**		** − 4.100**	**0.000**
Yes	215	55.8	0.83 (0.21)			78.73 (11.21)		
No	170	44.2	0.87 (0.20)			84.56 (10.97)		
Duration since diagnosis (years) Mean (SD) = 9.93 (7.54)				** − 0.218**	**0.000**		** − 0.207**	**0.001**
HbA1c (%) Mean (SD) = 10.31 (2.02)				− 0.062	0.242		** − 0.311**	**0.000**

### EQ-5D-3L in individuals with T2DM

As shown in Table 2, the pain/discomfort, anxiety/depression, mobility, usual activities, and self-care subscales of the EQ-5D-3L were 35.8%, 25.7%, 24.6%, 22.9%, and 12.9% among the study participants, respectively. The mean EQ-5D-3L and EQ-VAS scores were 0.85 (SD = 0.20) and 80.05 (SD = 0.22), respectively.

**Table 2:** EQ-5D-3L in individuals with T2DM (*n* = 385)

	No problem		Some problems		Extreme problems	
	N	%	N	%	N	%
Pain/discomfort	247	64.2	124	32.2	14	3.6
Anxiety/depression	286	74.3	84	21.8	15	3.9
Mobility	290	75.3	81	21.0	14	3.6
Usual activities	297	77.1	77	20.0	11	2.9
Self-care	335	87.0	41	10.6	9	2.3

### FOP, FOH, and SE in individuals with T2DM

Table 3 demonstrates that the scores of FOP-Q-SF HFSII and DESES were 26.84 (SD = 8.94), 71.67 (SD = 17.06), and 122.07 (SD = 38.31), respectively. In FOP-Q-SF, 23.1% of individuals scored ≥ 34 points. In HFSII, the score of HFSII-WS and HFSII-BS was 38.15 (SD = 10.57) and 33.52 (SD = 9.54), respectively. In DESES, the scores of medication blood glucose monitoring, diet, and exercise were 14.37 (SD = 5.05), 30.59 (SD = 10.40), 65.34 (SD = 23.29), and 11.77 (SD = 4.62), respectively.

**Table 3:** FOP, FOH, and SE in individuals with T2DM (*n* = 385)

Variable	Range of total scores	Total score (Mean [SD])	Mean score (Mean [SD])
**FOP**	12.00–56.00	26.84 (8.94)	2.24 (0.74)
**FOH**	33.00–115.00	71.67 (17.06)	2.17 (0.52)
Worry	18.00–64.00	38.15 (10.57)	2.12 (0.59)
Behavior	15.00–64.00	33.52 (9.54)	2.23 (0.64)
**SE**	22.00–200.00	122.07 (38.31)	6.10 (1.92)
Medication	2.00–20.00	14.37 (5.05)	7.19 (2.52)
Blood glucose Monitoring	5.00–50.00	30.59 (10.40)	6.12 (2.08)
Diet	11.00–110.00	65.34 (23.29)	5.94 (2.12)
Exercise	2.00–20.00	11.77 (4.62)	5.88 (2.31)

### Associated factors of HRQoL in individuals with T2DM

The univariate analysis identified a range of factors that were significantly associated with the HRQoL of the individuals with T2DM (Tables 1 and 4). Age (*r* =  − 0.262, *p* < 0.01), educational level (F = 9.181, *p* < 0.01), duration since diagnosis (r =  − 0.218, *p* < 0.01), and FOP (*r* =  − 0.275, *p* < 0.01) were significantly associated with EQ-5D-3L. Comorbid conditions (t =  − 4.100, *p* < 0.01), HbA1c (*r* =  − 0.311, *p* < 0.01), FOP (*r* =  − 0.128, *p* < 0.05), FOH (*r* =  − 0.295, *p* < 0.01), and SE (*r* = 0.246, *p* < 0.01) were significantly associated with EQ-VAS.

**Table 4:** Correlations between FOP, FOH, SE, and HRQoL in individuals with T2DM (*n* = 385)

	EQ-5D-3L	EQ-VAS	FOP	FOH	SE
EQ-5D-3L	1				
EQ-VAS	0.529^**^	1			
FOP	− 0.275^**^	− 0.128^*^	1		
FOH	− 0.073	− 0.295^**^	0.340^**^	1	
SE	0.089	0.246^**^	− 0.281^**^	− 0.172^*^	1

^*^p < 0.05, ^**^p < 0.01

The best-fit multiple linear regression model identified several factors that were significantly associated with EQ-5D-3L level, including age (95%CI [− 0.005, − 0.002], *p* < 0.01), FOP (95%CI [− 0.007, − 0.003], *p* < 0.01), duration since diagnosis (95%CI [− 0.007, − 0.002], *p* < 0.01), and educational level (95%CI [0.004, 0.062], *p* < 0.05) (Table 5). The best-fit multiple linear regression model identified several factors that were significantly associated with EQ-VAS level, including HbA1c (95%CI [− 1.724, − 0.316], *p* < 0.01), SE (95%CI [0.811, 4.571], *p* < 0.01), comorbid conditions (95%CI [1.147, 6.348], *p* < 0.01), and FOH (95%CI [− 6.765, − 0.818], *p* < 0.05) (Table 5).

**Table 5:** Associated factors of HRQoL in individuals with T2DM (*n* = 385)

Variable	*B*	*SE*–*B*	*β*	*t*	*P*	95% *CI*		VIF
						Low	Up	
**EQ-5D-3L**^a^								
Constant	1.129	0.075	-	15.045	0.000	0.982	1.277	-
Age (years)	− 0.003	0.001	− 0.226	− 3.901	0.000	− 0.005	− 0.002	1.481
FOP	− 0.005	0.001	− 0.213	− 4.146	0.000	− 0.007	− 0.003	1.160
Duration since diagnosis	− 0.004	0.001	− 0.161	− 3.090	0.002	− 0.007	− 0.002	1.187
Educational attainment^b^	0.033	0.015	0.118	2.234	0.026	0.004	0.062	1.221
**EQ-VAS**^a^								
Constant	67.154	5.521	-	12.163	0.000	56.273	78.035	-
HbA1c	− 1.020	0.357	− 0.184	− 2.857	0.005	− 1.724	− 0.316	1.153
SE	2.691	0.954	0.182	2.821	0.005	0.811	4.571	1.149
Comorbid conditions^b^	3.748	1.319	0.177	2.840	0.005	1.147	6.348	1.075
FOH	− 3.792	1.509	− 0.165	− 2.513	0.013	− 6.765	− 0.818	1.189

*Abbreviations*: *B* unstandardized coefficient beta, *SE–B* standard error of B, *β* Standardized coefficient beta, *95%CI* 95% confidence interval, *VIF* Variance inflation factor

^a^The R^2^ of EQ-5D-3L and EQ-VAS regression models were 33.5% and 40.8%, separately

^b^Educational attainment: 1 = Primary school, 2 = High school, 3 = College or above; Comorbid conditions: 1 = Yes, 2 = No

## Discussion

The HRQoL of individuals with T2DM directly reflects the degree of their physical health and spiritual happiness. Therefore, improving HRQoL is critical to maintaining the overall well-being of T2DM individuals. Our study used the EQ-5D-3L index and EQ-5D VAS to describe the participants’ HRQoL. We found that HRQoL was more likely to be impaired on the pain/discomfort dimension, which may be related to the increased burden of physical symptoms caused by poor blood sugar management with mean HbA1c levels higher than 10.30% among T2DM individuals. In addition, the level of EQ-5D-3L among T2DM individuals in our study was lower than those in the general community [35]. Compared with T2DM individuals in the community, hospitalized individuals are often newly diagnosed T2DM and (or) have advanced progression of T2DM; as many also have comorbidities or complications (e.g., peripheral vascular disease), they do not have sufficient experience or confidence to deal with the negative impacts of their disease on daily life, leading to decreased HRQoL levels [36]. Thus, paying attention to the HRQoL of hospitalized individuals in the endocrine department has considerably practical value.

FOP, which is an adaptive response that can become dysfunctional, is often experienced as the most severe distress symptom harmful to HRQoL. In participants with chronic diseases, FOP mediates their physical symptoms and HRQoL [8]. Our study showed that FOP was one of the main factors associated with participants’ HRQoL, where T2DM individuals with a higher level of FOP tended to have worse HRQoL. We found that 23.1% of T2DM individuals were suffering from psychological dysfunction, which might result in poor treatment compliance and glucose control [9]. Moreover, a chronic hyperglycemic state can induce a series of acute complications (e.g., ketoacidosis and hyperosmolar hyperglycemia syndrome) and chronic complications (e.g., macrovascular disease and microvascular disease), leading to physical discomfort and inconvenience in T2DM individuals [1, 2], possibly explaining their poor HRQoL. However, adverse reaction to HRQoL could be reduced by advanced intervention therapy on individuals’ FOP. As Frangou et al. [37] found, cognitive behavioral therapy–based psychological support had a significant positive impact on FOP, and the routine implementation of FOP support was called for in ovarian cancer patients. Therefore, medical professionals could follow the advanced clinical evidence and formulate a scientific plan to effectively assess and decrease FOP, thereby improving the HRQoL of T2DM individuals.

We also found that individuals with T2DM with a higher level of FOH could do harm to HRQoL. This result was similar to that of a European multicountry study, which showed that EQ-5D VAS scores of individuals with T2DM and detected hypoglycemic symptoms were significantly lower than individuals without FOH [38]. Due to T2DM, our participants’ daily life and work rhythm were disrupted [1, 2]; coupled with a higher level of FOH (having a profound impact on diabetes treatment and glucose control [39]) than previous studies [40, 41], the participants’ cognitive and psychological state were further threatened, which would result in their inability to maintain a good HRQoL. To improve HRQoL, it is necessary to strengthen knowledge and skills about distinguishing and preventing hypoglycemia by empowerment-based health education [42], peer coaches [43], and diabetes case management intervention [44] to avoid FOH among T2DM individuals.

It is worth noticing that few studies have elucidated the relationship between FOP and FOH. Our results showed that FOH was positively correlated with FOP. FOH caused by more frequent hypoglycemia was associated with the progression of T2DM becoming increasingly out of control [3, 4]. As diabetes progresses, individuals have to receive more treatment to balance blood sugar disorder, which would lead to an increased incidence of iatrogenic hypoglycemia [16, 17]. Meanwhile, individuals with FOH often engage in overcompensating behaviors, leading to poor FOP control in T2DM [15–17]. However, whether the coexistence of FOH and FOP synergistically amplify HRQoL damage requires further exploration in the future.

Consistent with previous research [21], SE was positively associated with HRQoL among individuals with T2DM, meaning that individuals with lower diabetes-management SE had worse HRQoL on average. Low SE can drain individuals; mental energy and exhaust their coping resources to deal with the stressors. T2DM, as a weighty stressor in one’s life, can cause a general decline in SE [45], which would cause individuals to lose confidence in managing blood sugar, leading to worsening physical symptoms and psychological distress [22]. These factors are additional reasons to decrease the HRQoL of T2DM individuals. Studies have showed that providing positive psychological assistance and advancing medical suggestions about glucose control for T2DM individuals are vital measures to enhance SE [17, 18]. Moreover, previous studies found that inventions (e.g., training in resilience skills) could build individuals’ confidence and increase their capacity to deal with stress factors, further improving SE and relieving FOP and FOH [46, 47]. In short, healthcare providers could improve HRQoL among T2DM individuals by providing positive psychological support and resilience skills training to promote SE.

To our knowledge, the state of blood glucose control among T2DM individuals can be detected using HbA1c [47]. Poor HbA1c often causes medical professionals to adjust the treatment regimens of individuals. Frequent adjustment could lead to hypoglycemia, which is a side-effect associated with some antidiabetic medications, and may also decrease treatment satisfaction [48]. These factors could induce a negative attitude when asked to follow medical orders. Hence, previous studies have shown that HbA1c level reflects the compliance of individuals with their diabetes treatment regimen, which is also an important factor affecting the HRQoL of individuals with diabetes [49].

Other variables, such as duration since diagnosed with T2DM, comorbid conditions, age, and educational attainment, had statistically significance impacts on HRQoL. Individuals with a longer duration since diagnosis of T2DM and more comorbid conditions (especially hypertension and hyperlipidemia) had more disease-related complications (e.g., diabetic foot and optic neuropathy), further affecting individuals’ HRQoL [50], as complications make it difficult for individuals to participate in normal daily activities. Elderly individuals with T2DM had worse HRQoL, which was consistent with Kang et al. [48] and may be due to the gradual decline of individuals’ physical function, intelligence, and other aspects with the increase of age, resulting in more discomfort. Individuals with higher educational level had better HRQoL, as seen in an American study [51]. T2DM individuals with a higher educational level may be more receptive to new things and be better at learning knowledge and skills to improve their condition in different ways.

### Limitations

Despite the supportive findings of the study, several limitations need to be acknowledged. Firstly, as the participants were only from five tertiary grade-A hospitals in Chongqing, China, the results might be difficult to generalize due to the particular sociodemographic characteristics. Furthermore, our study adopted a cross-sectional design, but a longitudinal design may better reflect the relationship between HRQoL and various associated factors. Finally, the R^2^ value of each multiple linear regression model was relatively small, and further studies are warranted to identify other associated factors of HRQoL.

## Conclusions

Our study found that T2DM patients’ HRQoL could further decrease with the increase of the patient’s age and duration since diagnosis. Low-level HbA1c reflects stable blood glucose control, which can indicate good HRQoL. Moreover, it is essential to note that individuals with T2DM suffer from varying degrees of negative effects of FOH and FOP for a long time, reducing HRQoL. Conversely, a high level of SE could effectively establish the confidence of individuals to deal with the progression of T2DM and improve HRQoL. Therefore, to improve HRQoL among T2DM individuals, we recommended that medical professionals follow the advanced clinical evidence, formulate a series of interventions (e.g., cognitive behavioral therapy–based psychological support, empowerment-based health education, and resilience skills training) to effectively decrease FOP and FOH, and enhance SE, thereby improving the HRQoL of T2DM individuals.

## Data Availability

The datasets used and/or analyzed during the current study are available from the corresponding author on reasonable request.

## Abbreviations

DMSES: Diabetes-Management Self-efficacy Scale
EQ-5D-3L: Three-level EuroQol Five Dimensions
EQ-VAS: EuroQol-Visual Analogue Scale
FOH: Fear of hypoglycemia
FOP: Fear of Progression
FOP-Q-SF: Fear of Progression Questionnaire-Short Form
HbA1c: Hemoglobin A1c
HFSII: Hypoglycemia Fear Survey II
HRQoL: Health-related quality of life
T2DM: Type 2 diabetes mellitus
QoL: Quality of life
SE: Self-efficacy
VAS: Visual analogue scale
